# Molecular epidemiology of *Enterocytozoon bieneusi* from foxes and raccoon dogs in the Henan and Hebei provinces in China

**DOI:** 10.1186/s12917-024-03883-6

**Published:** 2024-02-10

**Authors:** Minghui Chen, Haidong Wang, Xinmiao Li, Yunan Guo, Ying Lu, Liping Zheng, Guoqing Liang, Yuzhen Sui, Bukang Wang, Hongyu Dai, Haiju Dong, Longxian Zhang

**Affiliations:** College of Veterinary Medicine, Henan Agricultural University, Zhengzhou, Henan, 450046 China

**Keywords:** *Enterocytozoon Bieneusi*, Genotypes, Multilocus analysis, Foxes, Raccoon dogs

## Abstract

**Background:**

*Enterocytozoon bieneusi* is a zoonotic pathogen widely distributed in animals and humans. It can cause diarrhea and even death in immunocompromised hosts. Approximately 800 internal transcribed spacer (*ITS*) genotypes have been identified in *E. bieneusi*. Farmed foxes and raccoon dogs are closely associated to humans and might be the reservoir of *E. bieneusi* which is known to have zoonotic potential. However, there are only a few studies about *E. bieneusi* genotype identification and epidemiological survey in foxes and raccoon dogs in Henan and Hebei province. Thus, the present study investigated the infection rates and genotypes of *E. bieneusi* in farmed foxes and raccoon dogs in the Henan and Hebei provinces.

**Result:**

A total of 704 and 884 fecal specimens were collected from foxes and raccoon dogs, respectively. Nested PCR was conducted based on *ITS* of ribosomal RNA (*rRNA*), and then multilocus sequence typing (*MLST*) was conducted to analyze the genotypes. The result showed that infection rates of *E. bieneusi* in foxes and raccoon dogs were 18.32% and 5.54%, respectively. Ten *E. bieneusi* genotypes with zoonotic potential (*NCF2*, *NCF3*, *D*, *EbpC*, *CHN-DC1*, *SCF2*, *CHN-F1*, *Type IV*, *BEB4*, and *BEB6*) were identified in foxes and raccoon dogs. Totally 178 *ITS*-positive DNA specimens were identified from foxes and raccoon dogs and these specimens were then subjected to *MLST* analysis. In the *MLST* analysis, 12, 2, 7 and 8 genotypes were identified in at the mini-/ micro-satellite loci *MS1*, *MS3*, *MS4* and *MS7*, respectively. A total of 14 multilocus genotypes were generated using ClustalX 2.1 software. Overall, the present study evaluated the infection of *E. bieneusi* in foxes and raccoon dogs in the Henan and Hebei province, and investigated the zoonotic potential of the *E. bieneusi* in foxes and raccoon dogs.

**Conclusions:**

These findings expand the geographic distribution information of *E. bieneusi’* host in China and was helpful in preventing against the infection of *E. bieneusi* with zoonotic potential in foxes and raccoon dogs.

**Supplementary Information:**

The online version contains supplementary material available at 10.1186/s12917-024-03883-6.

## Background

Microsporidia are obligate intracellular parasites with hosts ranging from protists to mammals [1]. More than 200 genera and approximately 1,500 species of microsporidia have been identified, and 17 species causes the infection of human beings. *E. bieneusi* is responsible for more than 90% cases of human microsporidiosis infection [1–6]. Since its discovery in an acquired immune-deficiency syndrome patient in 1985, many genotypes have been identified [7, 8]. *E. bieneusi* could induce diarrhea or even death of the patient, but most of the patients infected with *E. bieneusi* only showed slightly dysbiosis or disruption of nutrient absorption [2, 9, 10].

More than 800 genotypes of *E. bieneusi* have been identified using polymorphism analysis of the internal transcribed spacer (*ITS*) region of the *rRNA* gene which belongs to 13 phylogenetic groups [2, 4, 11, 12]. More than 310 genotypes are included in Group 1 which are believed to infect both human and animals. *BEB4*, *BEB6*, *I*, and *J* are the dominant genotype in Group 2 which are found in ruminants, non-ruminant animals, and humans. Genotypes in Group 3–13 infect animals and showed little effect on public health [13]. Different groups of *E. bieneusi* genotypes display diverse zoonotic potential and host specificity [4]. Group 1 (e.g., genotype *D*, *Type IV*, and *EbpC*) is the largest group of *E. bieneusi* genotypes and can infect different kinds of animals with high adaptability to the environment [4, 14]. Most of Group2 members of *E. bieneusi*, e.g., genotype *I*, *J*, *BEB4*, and *BEB6* are the most common genotypes of *E. bieneusi* identified in sheep, goats, cattle, and deer [4, 6]. Most genotypes of *E. bieneusi* in groups 3–11 have a limited host range and thus pose a minor or unknown public health threat [4]. Nevertheless, the *ITS* genotyping method cannot fully reflect the genetic characteristics of *E. bieneusi* as it represents only a limited portion of the *E. bieneusi* genome (total length about 6 Mb) [15–17]. Multilocus sequence typing (*MLST*) is more discriminatory than *ITS* genotyping method by taking genetic polymorphisms of four mini- and microsatellites into account [8, 16, 18]. A higher genetic diversity was identified in *E. bieneusi* isolated from humans and animals using *MLST* analysis and several genetically isolating subgroups were formed within the *ITS* group 1 owing to their characteristics [19–21].

*E. bieneusi* can infect different animals and the zoonotic potential of *E. bieneusi* has been assessed in previous studies. Studies showed that the genotypes *D*, *EbpC*, and *IV* have considerable potential of cross-species infection due to their extremely broad host and geographic distribution [4]. Genotype *D* was first identified in raccoon dogs which raised the concerns regarding its potential for transmission to humans [22]. Other genotypes including *CHN-DC1*, *WildBoar3*, *CHN-R1*, *NCF2*, *CHN-F1*, *NCR2*, *NCR1*, *Korea-WL1*, *Korea-WL2*, *Korea-WL3*, *Korea-D*, *CHG1*, *Peru8*, *Type IV*, and *EbpA*, all belong to *ITS* group 1, were also found in raccoon dogs [11, 22–27]. Although the epidemiological investigation of *E. bieneusi* in foxes first began in 2003, it was not until 2014 when foxes were found to be infected with genotype *D* [28, 29]. Subsequently, genotypes *CHN-F*, *EbpC*, *Type IV*, *Peru8*, *NCF1*, *NCF2*, *NCF3*, *NCF4*, *NCF5*, *NCF6*, *NCF7*, *CHN-DC1*, *SDF1*, *SDF2*, *Hum-q1*, *HND-1*, and *C*, all belong to *ITS* group 1, were also identified in foxes [5, 22, 25, 27, 30, 31]. Previous studies showed that infection rate of *E. bieneusi* in raccoon dogs were 2.6–40.2% and were 7.7–30% in foxes [5, 11, 22–24, 26–31]. These findings suggest that *E. bieneusi* in raccoon dogs and foxes may be a source of *E. bieneusi* that causes the infection of humans.

However, there are only a few epidemiological studies on *E. bieneusi* in foxes and raccoon dogs in captivity worldwide. Thus, to further understand the genetic diversity of *E. bieneusi* in foxes and raccoon dogs, obtain geographic information, and compare the infection rates of *E. bieneusi* in different regions, the present study analyzed the infection rates and genotypes of *E. bieneusi* in farmed foxes and raccoon dogs in the Henan and Hebei provinces using *MLST*.

## Results and discussion

As shown in Table 1, a total of 178 *E. bieneusi-*positive samples (11.21%, 95% CI: 9.66–12.76) were identified via nested PCR based on the *ITS* locus in 1588 fecal samples from foxes and raccoon dogs and the total infection rate of *E. bieneusi* was similar with the total infection rate of *E. bieneusi* in farmed blue foxes and raccoon dogs was 12.6% in the Heilongjiang and Jilin Province [27], while was higher relative to the total infection rate of farmed blue foxes and raccoon dogs in Xinjiang China (2.7%) [32]. This indicated that prevalence of *E. bieneusi* was associated with geographic distribution of the animals. The infection rates of *E. bieneusi* were 8.65% (84/971), 7.81% (21/269), and 20.98% (73/348) in samples from Xinxiang city, Hebi city, and Changli city. The infection rate in samples from Changli city was significantly higher than that from Xinxiang city and Hebi city (*P* < 0.01). The infection rate in foxes was 18.32% (129/704), which was significantly higher relative to that in raccoon dogs (5.54%, 49/884) (*P* < 0.01). The infection rate of *E. bieneusi* in foxes and raccoon dogs in the present study was similar with previous findings in which *E. bieneusi* was detected in 16.4% (18/110) farmed blue foxes and 4.1% (2/49) raccoon dogs [27]. The infection rate in pre-weaned foxes (3.51%, 95% CI: 17.84–24.70) was lower than that in young (20.00%, 95% CI: 7.04–32.96) and adult foxes (21.27%, 95% CI: 17.84–24.70). The lower infection rate in pre-weaned foxes observed in the present study might be associated with the immune status and the antibodies contained in the colostrum, but the mechanism should be elucidated further. The infection rate in male foxes was slightly lower than that in female foxes which was different from previous study in which they found no significant difference in the infection rate of *E. bieneusi* between male and female foxes [33]. The infection rate in male raccoon dogs (8.17%, 95% CI: 5.67–10.67) was higher than that in female raccoon dogs (2.63%, 95% CI: 1.09–4.16), and this finding is in line with the results of previous studies [24, 26]. The differences observed in the infection rate of *E. bieneusi* in foxes and raccoon dogs of different gender in the present study maybe associated with sample size, different animal husbandry practice and animal welfare. Study demonstrated that no effective therapeutic method was available for the treatment of *E. bieneusi* [34]. This might be the reason why no significant difference in the infection rate between dewormed (dewormed with Avermectin) and non-dewormed farm animals was observed in the present study.

**Table 1:** Factors associated with the prevalence of *E. bieneusi* in farmed foxes and raccoon dogs in the Henan and Hebei provinces

Factor	Category	No. of positive animals/No. examined animals	% (95% CI)	OR (95% CI)	P value
Region	XinXiang	84/971	8.65 (6.88–10.42)	1	
	HeBi	21/269	7.81 (4.58–11.03)	0.89 (0.54–1.47)	0.66
	ChangLi	73/348	20.98 (16.68–25.28)	2.80 (1.99–3.94)	< 0.01
Host	Fox	129/704	18.32 (15.46–21.19)	1	
	Raccoon dog	49/884	5.54 (4.03–7.05)	0.26 (0.19–0.37)	< 0.01
Age^a^	Young	8/40	20.00 (7.04–32.96)	1	
	Pre-weaned	4/114	3.51(0.08–6.94)	0.15 (0.04–0.51)	< 0.01
	Adult	117/550	21.27 (17.84–24.70)	1.08 (0.49–2.41)	0.85
Gender^a^	Female	55/220	25.00 (19.23–30.77)	1	
	Male	74/484	15.29 (12.07–18.51)	0.54(0.37–0.80)	< 0.01
Age^b^	Young	7/209	3.35 (0.89–5.81)	1	
	Pre-weaned	12/208	5.77 (2.57–8.96)	1.77 (0.68–4.58)	0.24
	Adult	30/467	6.42 (4.19–8.16)	1.98(0.86–4.59)	0.10
Gender^b^	Female	11/419	2.63 (1.09–4.16)	1	
	Male	38/465	8.17 (5.67–10.67)	3.30(1.66–6.55)	< 0.01
Deworming condition	Dewormed	124/1147	10.81 (9.01–12.61)	1	
	Non-dewormed	54/441	12.24 (9.17–15.32)	1.15(0.82–1.62)	0.42
Total		178/1588	11.21 (9.66–12.76)		

OR, odds ratio: CI, confidence interval. ^a^ Samples from foxes. ^b^ Samples from raccoon dogs

As shown in Table 2, ten genotypes (*NCF2*, *NCF3*, *D*, *EbpC*, *CHN-DC1*, *SCF2*, *CHN-F1*, *Type IV*, *BEB4*, and *BEB6*) were identified by sequencing in the present study, among which genotype *NCF2* was the dominant one, and all genotypes identified in the present study were zoonotic [34]. The genotypes *NCF2*, *NCF3*, *D*, *CHN-DC1*, and *SCF2* has been identified in foxes previously [5, 22, 25, 27–31], but the genotypes *SCF2*, *CHN-F1*, and *BEB6* were first identified in foxes in the present study. Raccoon dogs has been reported to be infected with genotypes *NCF2*, *D*, and *Type IV* previously [11, 22–27], but the genotypes *NCF-3*, *EbpC*, *SCF-2*, *BEB4*, and *BEB6* were first identified in raccoon dogs in the present study (see Table 2). Among them, genotypes *BEB4* and *BEB6* belong to the *ITS* group 2 which have not been reported to be found in foxes and raccoon dogs. Previous studies identified genotype *BEB4* in cattle, yaks, pigs, humans, and non-human primates [4], while genotype *BEB6* was identified in cattle, sheep, goats, and humans [35, 36]. Thus, we hypothesized that genotypes *BEB4* and *BEB6* identified in foxes and raccoon dogs in the present study may be transmitted from cattle, because all the genotypes *BEB4* and *BEB6* identified in the present study were from the same farm which is close to a cattle farm. This transmission may be due to the contamination of the raw water by the feces of infected cattle in the farm nearby, but the prevalence of *E. bieneusi* in the cattle farm and raw water was not evaluated in the present study, therefore further study is still needed to clarify our hypothesis.

**Table 2:** Genotypes of *E. bieneusi* in farmed foxes and raccoon dogs in the Henan and Hebei provinces

Region	Host	No. of positive animals/ No. of examined animals	Genotype (No.)
XinXiang	Fox	61/412	NCF2 (3), NCF3 (2), D (26), CHN-F1 (26), CHN-DC1 (1), BEB6 (3),
	Raccoon dog	23/559	NCF2 (5), D (9), EbpC (1) Type IV; (3), BEB4 (2), BEB6 (3),
HeBi	Fox	10/130	NCF2 (7), SCF2 (3)
	Raccoon dog	11/139	NCF2 (8), NCF3 (1), SCF2 (2)
ChangLi	Fox	58/162	NCF2 (31), NCF3 (22), SCF2 (5)
	Raccoon dog	15/182	NCF2 (7), NCF3 (7), SCF2 (1)

**Fig. 1:**
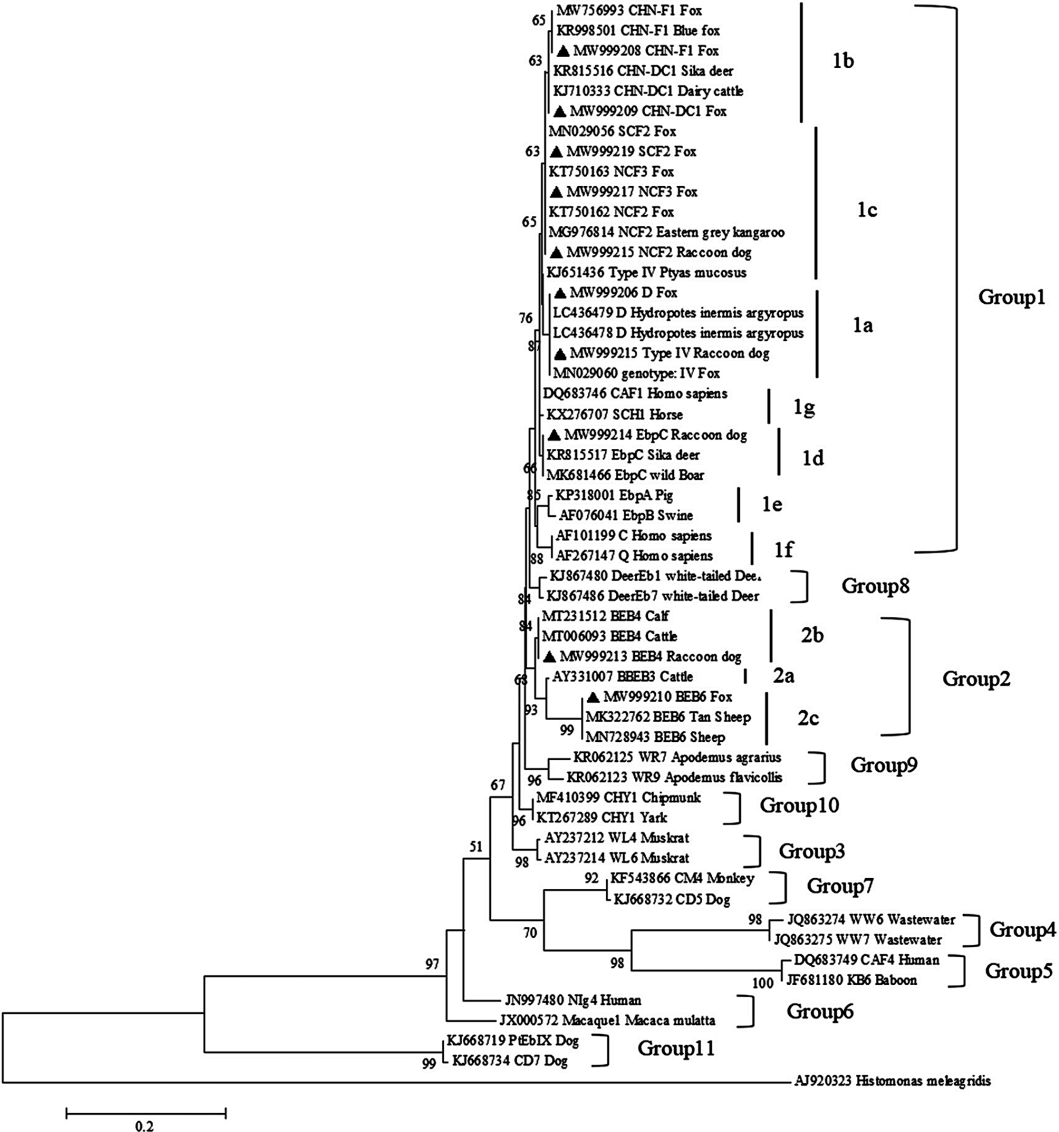
Phylogenetic relationships among *E. bieneusi* isolates inferred with a neighbor-joining analysis based on the ITS nucleotide sequences. The reliability of cluster formation was assessed by the bootstrap analysis with 1000 replicates, and the values generated greater than 50% are shown beside the nodes. The known ITS genotypes identified in the present study are indicated by black triangles

Phylogenetic analysis of the *ITS* loci showed that genotypes *NCF2*, *NCF3*, *D*, *EbpC*, *CHN-DC1*, *SCF2*, *CHN-F1*, and *Type IV* clustered into *ITS* group 1. Among them, genotypes *EbpC*, *Type IV*, and *D* are the most common genotypes of *E. bieneusi* that affect not only humans but also livestock and wild animals worldwide [4]. Although the genotypes *BEB4* and *BEB6* belong to *ITS* group 2 whose hosts are mostly ruminants, they may transmit to other hosts and lead to the infection of human beings [34]. Overall, these findings suggest that foxes and raccoon dogs may be potential sources of *E. bieneusi* infection in humans and other animals (Fig. 1).

At the *MS1*, *MS3*, *MS4* and *MS7* loci, 47 (43.93%), 74 (69.16%), 25 (23.36%) and 92 (85.98%) DNA specimens were amplified and sequenced successfully with 12, 2, 7, and 8 genotypes being identified, respectively. Eighteen multilocus genotypes (MLGs) were successfully amplified at all the five loci (*ITS*, *MS1*, *MS3*, *MS4*, and *MS7*), and 14 MLGs were formed (Table 3).

**Table 3:** Multilocus genotypes of 14 *E. bieneusi* isolates from foxes and raccoon dogs

Isolation	Multilocus genotype					
	ITS	MS1	MS3	MS4	MS7	MLG
A54	NCF2	Type1	Type2	Type5	Type1	MLG1
A834	D	Type1	Type2	Type5	Type1	MLG2
A101	D	Type2	Type1	Type4	Type3	MLG3
A422	CHN-F1	Type2	Type1	Type2	Type5	MLG4
A482	CHN-F1	Type2	Type1	Type2	Type5	MLG4
A518	CHN-F1	Type2	Type1	Type2	Type5	MLG4
A426	CHN-F1	Type2	Type2	Type2	Type5	MLG5
A454	CHN-F1	Type2	Type2	Type2	Type5	MLG5
A427	CHN-F1	Type2	Type2	Type5	Type2	MLG6
A442	CHN-F1	Type2	Type2	Type7	Type5	MLG7
A458	CHN-F1	Type2	Type1	Type1	Type5	MLG8
A460	CHN-F1	Type2	Type2	Type6	Type5	MLG9
B232	SCF2	Type2	Type1	Type6	Type2	MLG10
C83	NCF2	Type2	Type1	Type6	Type5	MLG11
A854	D	Type3	Type2	Type5	Type5	MLG12
A486	D	Type3	Type2	Type5	Type5	MLG12
B47	SCF2	Type3	Type2	Type3	Type5	MLG13
C117	NCF3	Type10	Type1	Type5	Type6	MLG14

**Fig. 2:**
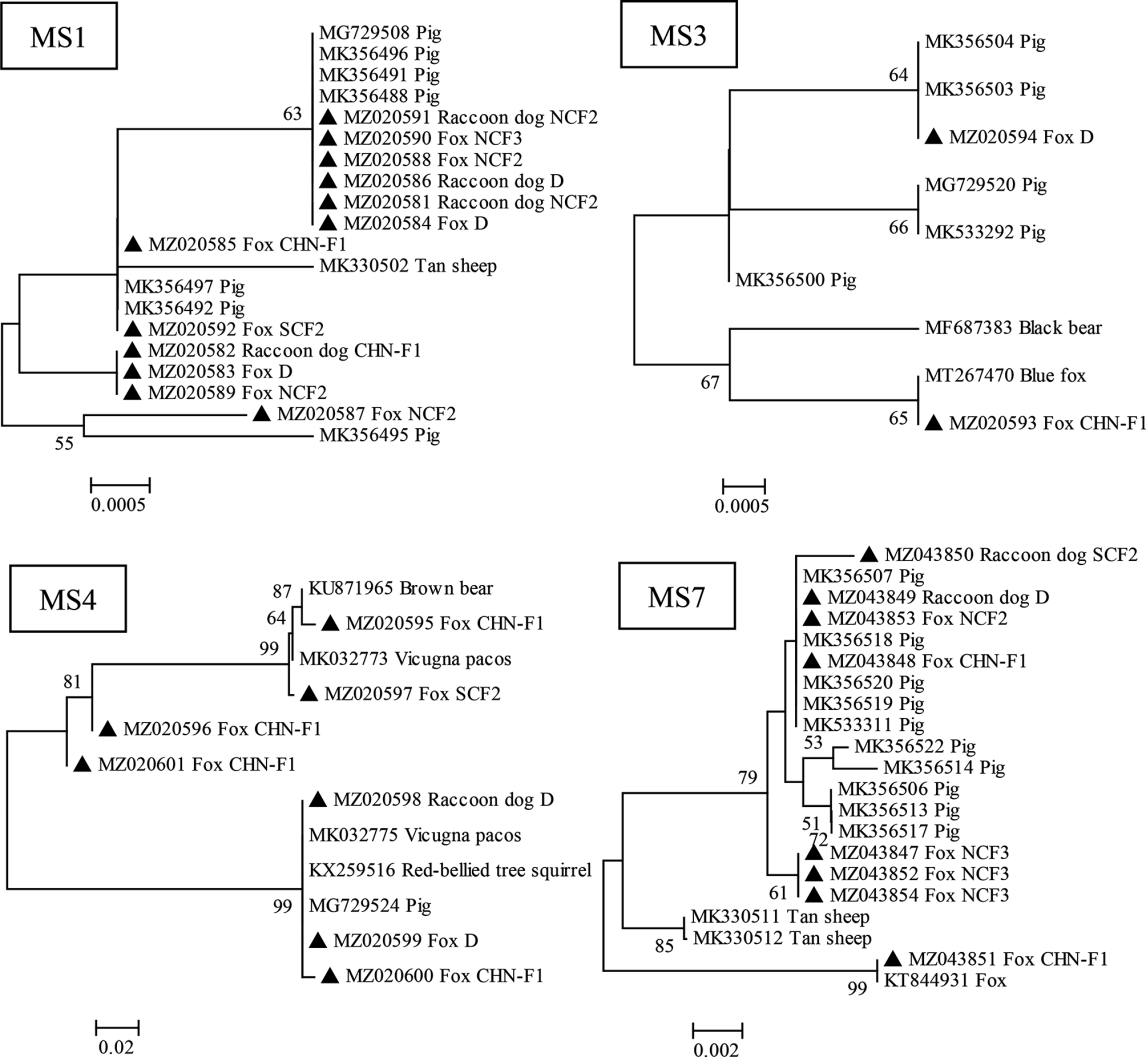
Phylogenetic relationships among *E. bieneusi* isolates inferred with a neighbor-joining analysis based on *MS1*, *MS3*, *MS4* and *MS7* locus, respectively. The reliability of cluster formation was assessed by the bootstrap analysis with 1000 replicates, and the values generated greater than 50% are shown beside the nodes. The types identified in the present study are indicated by black triangles

The phylogenetic analysis of microsatellite and microsatellite loci revealed that most of the *E. bieneusi* isolates from foxes and raccoon dogs were clustered together with the isolates from pigs and showed a close genetic match (Fig. 2). A few *E. bieneusi* isolates found in the present study were closest matched with the isolates from bear, Vicugna pacos, and squirrel and non from humans (Fig. 2), which is consistent with results of a previous study [18].

The findings of in the present study enrich the knowledge on the genetic diversity of *E. bieneusi* in foxes and raccoon dogs and performed the epidemiological investigation of *E. bieneusi* in foxes and raccoon dogs in the Henan Province and Hebei Province which has not been done in China. Currently, little information is available about the epidemiology of *E. bieneusi* in wild foxes and raccoon dogs; thus, wild species should be the focused in future studies.

## Conclusion

In the present study, differences in the infection rates of *E. bieneusi* in foxes and raccoon dogs were assessed by region, breed, age, sex, and deworming condition. Ten zoonotic *E. bieneusi* genotypes (i.e., *NCF2*, *NCF3*, *D*, *EbpC*, *CHN-DC1*, *SCF2*, *CHN-F1*, *Type IV*, *BEB4*, and *BEB6*) were identified, and a total of 14 *MLGs* were formed. Findings of the present study are benefit for the control and prevention of *E. bieneusi* infection in foxes and raccoon dogs.

## Methods

### Sample collection

Fresh fecal samples were collected from the rectum of foxes and raccoon dogs using disposable chlorinated polyethylene (CPE) gloves. Then specimens were placed in an ice-cold container and transported to the laboratory immediately. Half of the fecal samples were stored at 4 °C for DNA extraction, and the remaining samples were soaked in 2.5% potassium dichromate and stored at -20℃. A total of 1588 samples were collected between June and December 2020 from eight farms in Henan and Hebei province and full name of the farms were listed in table S1 in the supplementary file. The detailed information regarding sample collection was presented in Table 1.

### DNA extraction

Genomic DNA was extracted using the Stool DNA Kit (Omega Bio-Tek Inc., Norcross, GA, USA) according to the manufacturer’s instruction and the isolated DNA was stored at -20℃.

### PCR amplification and MLST

Infection of *E. bieneusi* were evaluated by nest PCR assay based on *ITS* locus, and the primers used in the present study has been described in our previous study [37]. The *ITS*-positive samples were selected based on *ITS* genotype, region, breed, age, and sex, and then were subjected to *MLST* analysis at the *MS1*, *MS3*, *MS4*, and *MS7* loci. The primers and annealing temperatures used in the *MLST* analysis of the present study were described previously [14]. The secondary PCR products were visualized by 1.5% agarose gel electrophoresis (containing 1 × 10^− 5^ DNA Green).

### Sequencing and phylogenetic analysis

The *ITS* positive secondary PCR products were sent to SinoGenoMax Biotechnology Co., Ltd. (Beijing, China) for sequencing and sequences obtained were aligned with reference sequences downloaded from the GenBank (http://blast.ncbi.nlm.nih.gov) using Clustal X 2.13 (http://www.clustal.org/) to confirm different species or genotypes.

To determine the phylogenetic relationships among the detected genotypes, neighbor-joining trees were constructed using the MEGA VII program (www.megasoftware.net) based on evolutionary distances calculated with the Kimura 2-parameter model. The reliability of these trees was assessed via bootstrap analysis of 1000 replicates.

### Statistical analysis

Significant differences in the prevalence of *E. bieneusi* among farmed foxes and raccoon dogs of different region, breed, age, sex, and deworming condition were analyzed using the chi-square test using SPSS version 26.0 (IBM Corporation, Armonk, NY, USA). Significant was defined at *P* < 0.05 and extremely significant defined at *P* < 0.01. The 95% confidence intervals (CIs) and odds ratios (ORs) were measured using SPSS version 26.0 (IBM Corporation, Armonk, NY, USA).

### Electronic supplementary material

Below is the link to the electronic supplementary material.

Supplementary Table S1: Names of the farms where the samples were collected

## Data Availability

The representative nucleotide sequences (ITS, MS1, MS3, MS4, and MS7) obtained in the present study are available in the [GenBank] repository, [https://submit.ncbi.nlm.nih.gov/]. The accession number of representative sequences are MW999206 - MW999220, MZ020581 - MZ020592, MZ020593 - MZ020594, MZ020595 - MZ020601, and MZ043847 - MZ043854, respectively.

## Abbreviations

ITS: internal transcribed spacer
MLST: multilocus sequence typing
MLG: multilocus genotype
