# Supplementary material for: Molecular epidemiology of Enterocytozoon bieneusi from foxes and raccoon dogs in the Henan and Hebei provinces in China
Source: BMC Vet Res. 2024 Feb 10;20:53. doi: 10.1186/s12917-024-03883-6 (PMC10858577; doi:10.1186/s12917-024-03883-6)
Supplement: Supplementary file 1 — Supplementary Table S1: Names of the farms where the samples were collected [file 12917_2024_3883_MOESM1_ESM.docx]

Table S1 Names of the farms where the samples were collected

| City | Name of the farm |
| --- | --- |
| Changli city | Xinji Farm |
|  | Xinzhuang Farm |
|  | East Tengyuan Farm |
| Hebei city | Qixian Farm |
|  | Damazhuang Farm |
| Xinxiang city | Yanjin Farm |
|  | Mazhuang Farm |
|  | Sizhai Farm |
